# Does the digital economy promote “innovation and entrepreneurship” in rural tourism in China?

**DOI:** 10.3389/fpsyg.2022.979027

**Published:** 2022-10-12

**Authors:** Gen Nian Tang, Fei Ren, Jie Zhou

**Affiliations:** ^1^School of Economics, Zhejiang University of Technology, Hang Zhou, China; ^2^School of Zhijiang, Zhejiang University of Technology, Hang Zhou, China; ^3^School of Marxism, Zhejiang Chinese Medical University, Hangzhou, China

**Keywords:** entrepreneurship, digital economy, product innovation, rural, tourism

## Abstract

This paper focuses on the impact of the “Digital Economy” on rural entrepreneurship. Unlike previous literature, the perspective of this paper focuses on a specific industry—tourism—and identifies a new mediating mechanism by which the “Digital Economy” affects rural tourism entrepreneurship—the promotion of innovation. The paper further clarifies the fact that the “Rural Digital Economy Index,” which is a dimension of the Digital Economy Indicator System, is the key to the mechanism of action. Theoretically, first, through a literature review, this paper provides a rationale for the “Digital Economy” to promote innovation behavior by reducing the cost of innovation. Second, using a product matching model, this paper argues that a rural tourism market characterized by innovation can stimulate more entrepreneurship. Empirically, using a sample of 150 counties in the Yangtze River Delta region of China, this paper argues that the higher the digitalization index of a county’s rural economy, the more national model villages and towns this county has (all of which include product innovation in the selection criteria) and more tourism entrepreneurial activities. Econometric methods such as endogeneity, spatial econometric regression, and sensitivity analysis proved the findings robust. Our recommendation is that the Chinese government could focus on improving the innovation environment for rural residents in the future, so that entrepreneurial activities will be spontaneously stimulated by market mechanisms.

## Introduction

The Chinese government has proposed to develop “Digital Economy” in rural areas during the 14th Five-Year Plan (2021–2025). Conceptually, the “Digital Economy” has a broader connotation than “Digital Technology.” Developing “Digital Technology” refers to the Chinese government’s provision of Internet, mobile Internet, e-commerce and other infrastructure as well as online trading platforms in rural areas. Developing “Digital Economy” means that based on the development of digital infrastructure construction (measured by the “Digital Infrastructure Index”), the Chinese government promotes the integration of data elements into rural production processes (measured by the “Rural Economy Digitalization Index”), the digital products and services into farmers’ lives, and the digital thinking into rural government services (measured by the “Rural Governance Digital Index”), so as to provide digital impetus to achieve the comprehensive revitalization of rural areas. On the other hand, as an important solution to the problem of rural poverty, “mass entrepreneurship and innovation” is a long-term national policy of the Chinese government to promote rural-economic vitality. Entrepreneurship can increase the income of rural entrepreneurs, create more jobs in rural communities, and increase the economic growth rate in lagging rural areas (Stephens et al., 2013; Blattman et al., 2014; Dong et al., 2021). Therefore, this paper focuses on whether the digital economy helps to stimulate entrepreneurial activities in rural China. Further, this paper attempts to answer which specific Indicator of the “Digital Economy” (Digital Infrastructure Index, “Rural Economy Digitalization Index” or “Rural Governance Digitalization Index”) is more conducive to stimulating entrepreneurship and how to stimulate entrepreneurship, with the aim of improving the science and effectiveness of the government’s future supporting policies.

Many scholars have focused on the relationship between the “Digital Economy” (or rather, “Digital Technology”) and entrepreneurship. Cumming and Johan (2010) suggest that the Internet leads to a clustering of rural entrepreneurial activity. Beck et al. (2018) discusses the impact of the mobile Internet on entrepreneurship, an article unique in that it is calibrated using a general equilibrium model. Barba-Sánchez et al. (2021) concluded that the information technology capabilities of entrepreneurs have a positive relationship with entrepreneurship. Deller et al. (2022) studied the impact of broadband speed on local new venture creation, this article specifically divided broadband speed into several dimensions and found the indicators that most affect entrepreneurial outcomes, this line of research provides insights for our study. Tan and Li (2022) proposed that Internet technology has a greater effect on promoting entrepreneurship in rural areas of China than in cities, and the mechanism of action is to improve rural entrepreneurs’ access to information and financing. Wang et al. (2019) proposed another mechanism by which the Internet influences rural entrepreneurial activity—social networks. Barnett et al. (2019) explored that the popularity of smartphones affects rural entrepreneurial activity and the mechanisms of action are information accessibility and social networks. Bogoviz et al. (2017) argues that there are several factors that moderate the impact of digital technology on entrepreneurial activity.

In summary, the academic progress in this field exhibits the following characteristics: (1) Regarding the object of research, attention has been paid both to the impact of technology itself and of different dimensions of technology. (2) Regarding the research methodology, some scholars have started to use general equilibrium analysis of economics as a research paradigm. (3) Regarding the research focus, scholars have increasingly focused on the mechanisms of the Digital Economy ‘s effect on entrepreneurial activity.

Unlike these literatures, the marginal contributions and innovations of this paper are as follows: First, this paper focuses on entrepreneurial activity in one specific sector in rural China—tourism, which accounts for one-third of all tourism revenue in China and is one of the most important industries in rural areas. Limiting the relationship between the “Digital Economy” and entrepreneurship to one industry specifically helps us to identify new mechanisms of action, namely, the “Digital Economy” promotes innovation in rural tourism products, and a tourism market characterized by innovation in turn promotes more entrepreneurial activity.

Second, this paper explicitly uses “Digital Economy” as an explanatory variable, which, as mentioned earlier, is much more encompassing. The data is derived from an official survey conducted by Peking University, and it contains 4 primary indicator, 13 secondary indicators, and 39 tertiary indicators, based on county-level administrative districts (administrative districts below the urban level and dominated by the township economy). Based on the needs of the study, this paper uses 3 of 4 first-level indicators (shown in Table 1), which portray a county’s investment in digital infrastructure, a county operator’s understanding and use of digital technology, and a county’s level of digital governance, respectively. We focus on which of these indicators can work through intermediary mechanisms.

**Table 1:** Dimensions of the digital economy.

	Primary	Descriptions
Digital Economy	Digital Infrastructure Index(“DII” in short)	Level of government investment in Internet, mobile Internet, big data platforms, Internet of things, and other related facilities.
	Rural Economy Digitalization Index (“REDI” in short)	Entrepreneurs’ understanding of digital technology; entrepreneurs’ use of data in production and marketing; digital transformation of industry chain business models.
	Rural Governance Digital Index (“RGDI” in short)	Government use of digital technology in service delivery; government use of digital technology in managing markets

Finally, based on the clear market scope, this paper uses the general equilibrium analysis of neoclassical economics as a research paradigm to provide explicit mathematical expressions for the causal relationship between the “Digital Economy” and entrepreneurship.

The subsequent content is organized as follows: The second part is the theoretical analysis, which consists of two subsections. The first subsection gives the theoretical basis for the “Digital Economy” to promote product innovation in rural tourism by means of a literature review. The second subsection uses the product matching model from industrial organization theory to argue that a rural tourism market characterized by differentiated innovation stimulates more entrepreneurial activities.

The third section is an empirical analysis that uses an econometric model to test the relationship between the “Digital Economy” and product innovation, as well as the “Digital Economy” and entrepreneurial activities, respectively. Among them, regarding product innovation, this paper creatively uses the proxy variable measure. This chapter includes four subsections. First, we introduce the sample of the study, the values of the explanatory and explanatory variables, and the descriptive statistics of the sample. Second, we present the econometric model and analyzes the computational results. Third, we discuss the endogeneity problem of the explanatory variables and gives solutions. At last, we discuss a series of robustness issues.

The fourth section concludes the study. First, we give the most central conclusion of the paper. Second, we make policy recommendations based on the conclusions. Finally, we illustrate where the paper can be improved and where future research can be directed.

## Theoretical analysis

Why do people choose to start a business in a specific industry (tourism) in a specific geographic area (rural)? According to microeconomics, an important pull factor is the ability of the industry to provide entrepreneurs with a higher return on investment, or excess profits (Falco and Haywood, 2016). When tourist preferences are heterogeneous and monopoly profits come from operators offering differentiated products through innovation, monopoly profits will further encourage more rural residents to enter tourism through entrepreneurship, when the market is characterized by product differentiation. However, rural tourism in China has been characterized by product homogeneity for a long time, which has led to two scenarios: on the one hand, Chinese tourists’ preferences are not met and the consumer market is not made bigger and stronger; on the other hand, price competition among operators is exceptionally stimulating and operating profits are meager. As a result, product homogeneity has reduced the dynamics of the rural tourism market and constrained more entrepreneurial behavior.

### Digital economy and rural tourism product innovation

#### One of the reasons for the homogenization of rural tourism in China: The cost of innovation

Scholars such as Xu et al. (2013), Ding and Ma (2018), and Xu et al. (2021) have successively explored the phenomenon of homogenization of rural tourism in China and its causes. In addition, a large number of studies published in Chinese domestic academic journals also provide explanations for the homogenization phenomenon, which, in summary, include the following: (1) Lack of information channels makes it difficult for rural tourism entrepreneurs to access the consumption preferences of urban tourists. (2) Lack of risk protection mechanisms, and the inability of financial institutions to access entrepreneurs’ credit information and their reluctance to provide financial support. (3) Lack of coordination mechanism; tourism behavior contains multiple links, and product innovation in a single link is difficult to obtain cooperation from other links in the industry chain. (4) The Chinese government’s habit of setting entrepreneurial role models indirectly leads to product homogenization. Solving the above problems requires a new market environment and institutional environment, which is unlikely to be changed by entrepreneurs alone. When the cost of innovation in a given environment is high, entrepreneurs are reluctant to innovate according to Arrow’s (1962) model of innovation incentives.

#### The emergence of the “digital economy”: Reducing the cost of innovation

From the development practice, it is clear that the “Digital Economy” as a new economic form effectively solves the above mentioned factors that lead to product homogenization, and thus reduces the cost of innovation. Taking the practice in China as an example. For factor (1), the combination of the Internet and technologies such as big data and cloud computing can achieve an accurate portrait of both supply and demand, solve the problem of asymmetric market information, and increase the probability of successful innovation. For factor (2), digital finance meets the financial needs of rural industries, solves the problems of financing difficulties and high financing costs for rural entrepreneurs, and reduces the innovation risks of entrepreneurs. For factor (3), the “Digital Economy” has given rise to the platform economy, a new business model (Spulber, 2019), which promotes entrepreneurs located in different parts of the industry chain to cooperate under a unified rule and framework and encourages collaborative innovation. For factor (4), the “Digital Economy” improves the government’s governance capacity and governance efficiency, strengthens the consultation and interaction between policymakers and stakeholders, and improves the monitoring mechanism so that innovative behavior is protected.

Many academic results provide a theoretical basis for the inference that the “Digital Economy” can reduce the cost of innovation. Asghari and Gedeon (2010) proposed that information technology reduces the transaction and administrative costs of firms and enhances customer communication. Goldfarb and Tucker (2019) argue that digital technologies such as the Internet can reduce costs in five areas, including search costs, replication costs, transportation costs, tracking costs, and verification costs. Among them, reducing search costs can improve the efficiency of information communication and organization; reducing replication costs can help companies to innovate in new product development. Bhimani et al. (2019) proposed that social media reduces knowledge flow costs and knowledge management costs and drives customer-centric innovation behavior. Urbinati et al. (2020) similarly suggested that digital technologies can reduce the cost of managing knowledge and help firms develop and nurture open innovation. Moreover, since the key to successful innovation is managing information and knowledge (Barba-Sánchez et al., 2018), which is carried in data, digitally driven innovation (DDI) becomes a new innovation strategy (Saura et al., 2021).

#### Evidence on innovation costs and innovation behavior

Moser (2005) analyzes the impact of patent law on innovation and suggests that innovation only occurs when profits can be expected. Simons and Astebro (2010) argue that whether inventors commercialize their inventions depends on profits. Although these two papers do not explicitly present costs, costs are internalized in profits since profits are composed of benefits and costs. Another related paper comes from Cucculelli and Ermini (2013), where the authors suggest that entrepreneurs willing to innovate a product need to be risk-averse due to the presence of innovation costs that lead to uncertainty of returns.

### Innovation promotes entrepreneurship

We use the general equilibrium research paradigm of economics to argue for the relationship between rural tourism innovation and rural tourism entrepreneurship. First, we construct a virtual tourism market in a rural area and make the following assumptions: (1) the number of tourists is N and has heterogeneous preferences; (2) the number of entrepreneurs already in the market is M. Entrepreneurs seek to maximize profits and, influenced by the “Digital Economy” mentioned above, each entrepreneur is willing to offer one (and only one) differentiated product through innovation.

Second, we use a product matching model to describe the relationship between supply and consumption in the market. The product matching model is derived from Hotelling’s one-dimensional linear model, in which N tourists are uniformly distributed in a one-dimensional space and each tourist occupies a specific location ${\bar{r}}_{i}$, and the model uses different locations to denote different preferences. M entrepreneurs are also uniformly distributed in a one-dimensional space (which can also be expressed as M products uniformly distributed in a one-dimensional space), and each entrepreneur (hereafter uniformly “each product”) occupies a specific position $r_{i}$, and the model uses different positions to represent different product characteristics.

**Figure 1:**
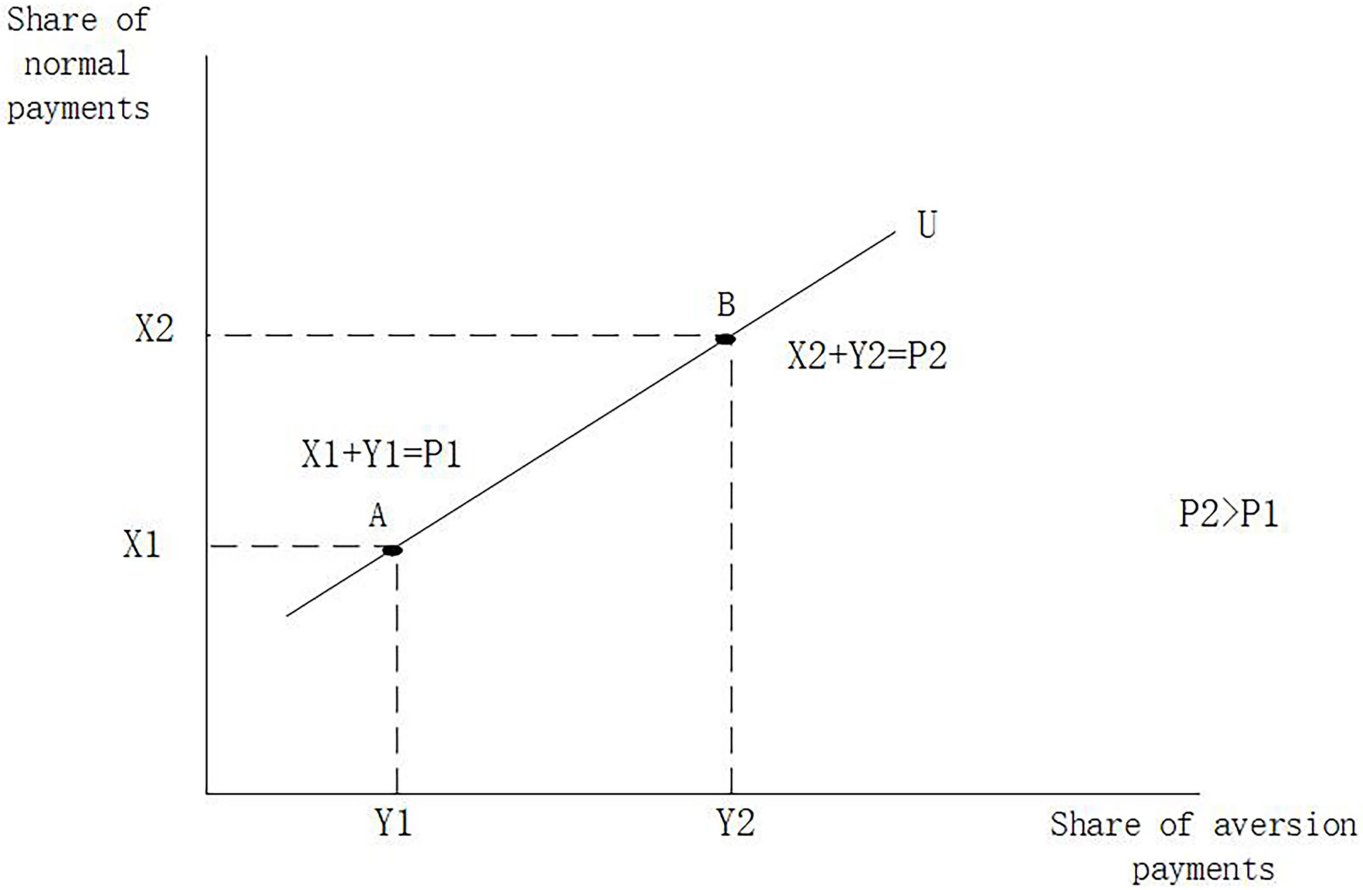
Note: In the above figure, *P*1 at point A corresponds to the actual price paid by the tourist, but due to the incomplete match between the tourist product characteristics and the tourist preferences, only the amount paid for *X*1 brings value return, while the amount paid for *Y*1 brings a non-positive value return. If tourists’ preferences and tourism product characteristics match perfectly, at point A, the expenditure of *P*1 should be fully compensated by the value return of *X*2, but there is a deviation, and the price of *P*2 must be paid to obtain the value return of *X*2, which means more payments that cannot obtain the value return. If the consumer makes a choice based on utility, *A* and *B* are equivalent, so we convert the consumer’s utility at point *A* to the utility at point *B*.

When ${\bar{r}}_{i}=r_{i}$, the visitor’s preference matches exactly with the characteristics of product i, and the visitor will receive 100% of the value return by paying the price of $p_{i}$. When ${\bar{r}}_{i}\neq r_{i}$, the visitor’s preference does not match the characteristics of product i, and the visitor will receive less than 100% of the value return by paying a price of $p_{i}$. Here a utility conversion is performed where we convert spending $p_{i}$ to receive a value return less than 100% to spending more than $p_{i}$ to receive a value return equal to 100%; the conversion does not change the visitor utility. The basis for such a conversion is that a product cannot be completely inconsistent with tourists’ preferences, and in the total payment, we regard the payment that meets preferences as normal goods and the payment that does not meet preferences as aversive goods, then the slope of the utility curve is positive and the utility at low payments can be equal to the utility at high payments. At this point, the utility of visitors at each position in the model is the same, and the consumption decision depends on the price paid, which can be represented by a mathematical formula. We give a more detailed explanation in graphical form in Figure 1. Consumers who do not match the product characteristics pay an additional price of $\sigma {\left|{\bar{r}}_{i}-r_{i}\right|}^{2}$ and receive 100% value in return. The consumer will choose the one with the minimum price paid between the two nearest products.

There is a position between two tourism products adjacent to each other in the model, and this position is the demarcation point of the market range of the two products. Because the model is one-dimensional, product i has a demarcation point in each of the left and right directions, respectively, and the range between the demarcation points is the market range of product i. We denote the demarcation point and the market range by $\left[{\bar{r}}_{i},{\bar{r}}_{i+1}\right]$. At the demarcation point, tourists consume the nearest product on the left side with equal payment to consume the nearest product on the right side, and for tourists, there is no difference between the two products. At the other locations, only one product pays the minimum price and is chosen by the tourist. Therefore, there exists


(1) $${\bar{r}}_{i}=\frac{p_{i}-p_{i-1}+\sigma \left(r_{i}^{2}-r_{i-1}^{2}\right)}{2\sigma \left(r_{i}-r_{i-1}\right)}$$

(2) $${\bar{r}}_{i+1}=\frac{p_{i+1}-p_{i}+\sigma \left(r_{i+1}^{2}-r_{i}^{2}\right)}{2\sigma \left(r_{i+1}-r_{i}\right)}$$

Under the assumption that each entrepreneur (or each product) seeks to maximize profit, the profit equation can be expressed as


(3) $$\pi_{i}=\overset{{\bar{r}}_{i+1}}{\underset{{\bar{r}}_{i}}{\int }}N\left(p_{i}-c\right)dr=N\left(p_{i}-c\right)\left({\bar{r}}_{i+1}-{\bar{r}}_{i}\right)$$

The *c* in the above equation represents the marginal cost of 1 unit of rural tourism product. We proceed to calculate the first-order partial derivative of $\pi_{i}$ with respect to $p_{i}$. Substitute Eqs. (1) and (2) into Eq. (3), we have


(4) $$p^{*}\left(M\right)=c+\frac{\sigma }{M^{2}}$$

Equation (4) shows that entrepreneurs price tourism products above the marginal cost of tourism products, which indicates that product innovation brings market power to entrepreneurs, and entrepreneurs can thus earn excess profits (or monopoly profits). The excess profit attracts more entrepreneurs to enter the tourism market, and new entrants also need to pay the innovation cost of F(θ), where θ is a measure of the level of development of the “Digital Economy” and $F^{\prime }\left(\theta \right)<0$. Therefore, the number of entrepreneurs in the tourism market at equilibrium can be expressed as [substituting equation (4) and F into Eq. (3)]


(5) $$M^{*}=\sqrt[{3}]{\frac{\sigma N}{F\left(\theta \right)}}$$

We can find that when the market reaches equilibrium, the number of entrepreneurship in the market is proportional to the number of tourists N, inversely proportional to the innovation cost F (θ), and proportional to the overpayment σ resulting from the mismatch of consumer preferences for product characteristics. The number of tourists and overpayments are not the focus of this paper; we are concerned with the fact that the “Digital Economy” can reduce the cost of innovation and thus facilitate the continuous entry of rural entrepreneurial agents.

In the theoretical analysis section, we establish a link between the “Digital Economy” and rural tourism entrepreneurship and use tourism product innovation as a mediating channel. The “Digital Economy” reduces innovation costs and promotes innovation, which in turn promotes entrepreneurial activity. We next test the linkage in the empirical analysis section.

## Empirical analysis

In this paper, 150 counties in the Yangtze River Delta region (YRD) consisting of Jiangsu (JS), Zhejiang (ZJ) and Anhui (AH) provinces, which share similar cultural attributes and institutional environments, are selected as the sample. In addition, after more than 10 years of integrated development, the economic levels of different sub-regions in the Yangtze River Delta tend to converge. Therefore, this helps us to identify the net effect of the “Digital Economy.”

### Data

#### Independent variable

The independent variable is the level of “Digital Economy” development of each county. As mentioned above, we used the County Digital Countryside Index (2018) published by New Rural Development Institute of Peking University in conjunction with Ali Research Institute. This data is a cross section of the 2018. We chose three of the primary indicators (“Digital Infrastructure Index”) (“DII” in short), “Rural Economy Digitalization Index” (“REDI” in short), and “Rural Governance Digitalization Index” (“RGDI” in short) as the values of the independent variables.

“Digital Infrastructure Index” mainly contains a measure of the level of rural information infrastructure, the level of digital financial infrastructure and the level of big data platforms, reflecting the level of government investment in the process of developing the “Digital Economy.” “Rural Economy Digitalization Index” mainly contains the digitalization level of enterprise production, the digitalization level of industrial chain, and the digital marketing level of enterprises, which reflects the understanding and application of digital technology by market operators. “Rural Governance Digital Index” includes the level of digitization of government services. Unlike the Digital Infrastructure Index, this index reflects whether the government has improved its governance efficiency through digitization.

#### Dependent variable

The dependent variable was used to describe the entrepreneurial performance of the entrepreneurs in each county. We have selected three representative entrepreneurial performances, which are: (1) Number of new startups influenced by the “Digital Economy” in a given period, (2) Number of entrepreneurial exits influenced by the “Digital Economy” in a given period, and (3) Average life cycle of exited startups influenced by the “Digital Economy.” Among them, we are most concerned about the indicator of the number of new startups.

The data comes from the National Enterprise Credit Information Public Disclosure System provided by the Chinese government. We searched for startups using “farmhouse” as a keyword—the Chinese name is “农家乐,” similar to the European “family hotel.” The “farmhouse” is a kind of small business that is widely found in rural areas and can provide food, accommodation, leisure and fun, etc. It is usually operated by rural residents using their own houses. The national enterprise credit information disclosure system can provide data on the “farmhouse” in each county, including the total number of farmhouses in a county at a given time, the start-up time of each farmhouse, the exit time of those that have been withdrawn, and the registered capital, address, and contact information of the farmhouse.

The digitalization of China’s rural areas can be traced back to 2008 when the Ministry of Agriculture proposed “Opinions on Accelerating the Promotion of Rural Informatization” and 2009 when the National Tourism Administration started the “Rural Smart Tourism.” In addition, considering that the Rural Digital Economy Index was released in 2018, we set the observation period of the sample from 2011 to 2020, which can completely cover the 12th and 13th Five-Year Plan periods of China. We calculate the values of three specific dependent variables: (1) Increase in entrepreneurship (“IIE” in short), the ratio of the average number of new “farmhouses “per year between 2011 and 2020 to the stock of “farmhouses” in 2018, using the ratio form to better control for industry. (2) Exit of entrepreneurship (“EOE” in short), the ratio of the average number of “farmhouses “exited each year between 2011 and 2020 to the stock of “farmhouses” in 2018. (3) Entrepreneurial life cycle (“ELC” in short), the average survival time of the exited farmhouses between 2011 and 2020.

#### Intermediate variables

The mediating variable is the level of differentiation of a county’s rural tourism product, which comes from innovation. Due to the lack of abundant data on firms at the rural level in China, we use three proxy variables to describe the level of differentiation. The first proxy variable is the number of “National One Village, One Product Model Villages” (leisure tourism category) owned by a county. The selection criteria for “National One Village, One Product Model Villages” (“OPMV” in short) include “having more new industries and new business models” and “having distinctive brands,” which can indirectly reflect regional innovation performance. The second proxy variable is the number of “China’s Beautiful Leisure Villages” (“CBLV” in short) owned by a county. The selection criteria for “China’s Beautiful Leisure Villages” include “having a variety of tourism products” and “products recognized by consumers,” which can directly reflect regional innovation performance. The third proxy variable is the number of “National Key Villages of Rural Tourism” owned by a county. The selection criteria for “National Rural Tourism Key Villages” (“RTKV” in short) include a “well-developed tourism product system” and a “unique tourism theme,” which is a direct indicator of regional innovation performance. It is important to note that the second and third proxies are provided by different departments of the Chinese government and can be used as references. A more detailed description can be found in Table 2. In addition, we provide statistical values for the independent, dependent, and mediating variables at the provincial level and at the YRD level in Table 3.

**Table 2:** Basic information and selection criteria for the three types of selected lists (excerpt).

Government sector	Name	Time (group)	Num	Criteria for selection (excerpt)
Ministry of Agriculture	National One Village, One Product Model Villages	2011–2020 (10 groups)	3,387	1. Promote product innovation through industrial integration 2. Already have a new product system and new business model 3. Industrial development has increased farmers’ income
Ministry of Agriculture	China’s Beautiful Leisure Villages	2010–2016; 2017–2021 (10 groups)	–^*^	1. Products widely praised by consumers. 2. There are various types of tourism products, such as leisure agriculture park, B&B, etc. 3. A number of travel brands have been created
National Tourism Bureau	National Key Villages of Rural Tourism	2019–2021 (3 groups)	1,199	1. High quality and variety of tourism products 2. Tourism products with a clear theme 3. Villagers get higher income because of tourism

* The official website of China’s Ministry of Rural Affairs can only be consulted for the qualified list of “China’s Beautiful Leisure Villages” from 2011 to 2016.

**Table 3:** Statistical values of independent, dependent, and mediating variables (proxy variables) at the YRD level and at the provincial level.

Variable	Name		YRD	JS	ZJ	AH
Independent	DII	Mean	80.48	78.12	87.92	75.70
		S.D	12.65	13.76	11.90	9.43
	REDI	Mean	53.13	55.84	58.74	46.75
		S.D	12.02	12.26	10.98	9.62
	RGDI	Mean	70.15	74.22	87.65	52.99
		S.D	19.74	13.31	11.22	13.53
Dependent	IIE	Mean	75.86	77.09	78.57	72.81
		S.D	12.56	10.35	9.88	15.10
	EOE	Mean	13.16	13.27	13.22	13.04
		S.D	5.36	4.86	6.81	6.39
	ELC(Month)	Mean	75.95	78.09	68.30	81.01
		S.D	14.16	13.51	15.96	9.73
Mediating	OPMV	Mean	0.32	0.28	0.43	0.24
		S.D	0.62	0.61	0.64	0.62
	CBLV	Mean	1.28	1.73	0.98	1.24
		S.D	1.25	1.55	1.10	1.09
	RTKV	Mean	0.19	0.15	0.21	0.18
		S.D	0.39	0.36	0.41	0.38
	Number of counties		150	38	51	61

Yangtze River Delta data does not include Shanghai sample; Beautiful Country 2010–2016 only qualified list.

### Digital economy and entrepreneurial activity

The baseline model is shown below, as we focus more on new entrepreneurship, the dependent variable on the left side of the equation is the increase in entrepreneurship (IIE). The other two dependent variables need to be changed in form and are not repeated here.


$$\frac{\Delta IIE_{C}}{L_{C,2018}}=\beta \;Digital_{c}+\Gamma X_{c,2018}+\mu_{i}+\varepsilon_{c}$$

$\frac{\Delta IIE_{C}}{L_{C,2018}}$ represents the ratio of the average annual number of new “Nongjiale” in a county from 2011 to 2020 to the stock of “Nongjiale” in 2018, and the subscript c indicates a specific county. $Digital_{c}$ is a set of digital economy variables, using digital infrastructure index (“DII” in short), rural economy digital index (“REDI” in short) and rural governance digital index (“RGDI” in short), respectively. $X_{c,2018}$ is a set of control variables, including (1) the number of national 4A and 5A scenic spots in the county, in order to control the high-quality tourism resources; (2) GDP, in order to control the level of regional economy; (3) government financial expenditure, in order to control government behavior; (4) administrative area, in order to control the scale; (5) the area of facility agriculture, in order to control the level of agricultural development; (6) the number of service industry employees, in order to control the level of service industry development; (7) the topographic index, in order to control the possible differences of natural landscape; (8) the bank deposit balance, in order to control the capital abundance of the county; (9) the number of industrial enterprises above the scale, in order to control the characteristics of scale economy. In addition, we added provincial fixed effects, in order to control the tourism policies at the provincial level. Table 4 shows the baseline regression results.

**Table 4:** Baseline regression results for the digital economy and entrepreneurship.

	A:IIE			B:EOE			C:ELC		
DII	0.433^***^ (0.089)			0.037 (0.058)			0.086 (0.08)		
REDI		0.439^***^ (0.109)			−0.324^***^ (0.058)			0.211^*^ (0.099)	
RGDI			0.277^**^ (0.086)			−0.036 (0.067)			−0.009 (0.071)
Control	Yes	Yes	Yes	Yes	Yes	Yes	Yes	Yes	Yes
*F*	5.11	4.89	3.17	3.35	9.30	1.78	3.33	3.71	3.36
Prob > *F*	0.000	0.000	0.001	0.000	0.000	0.036	0.000	0.000	0.000
*R*-squared	0.251	0.241	0.216	0.264	0.366	0.114	0.209	0.229	0.213
obs	150	150	150	150	150	150	150	150	150

* indicates significant at the 0.05 level,

** indicates significant at the 0.01 level.

*** indicates significant at the 0.001 level.

1. The values of the first two dependent variables are multiplied by 100 when entering the regression. 2. All control variables such as gross regional product, resident deposits, fiscal expenditure, administrative area, and number of industrial enterprises above the scale are taken as logarithms.

In column A of Table 4, all three rural digital economy indicators show a positive and significant relationship with “Increased In Entrepreneurship” (IIE). The economic implication is that the higher the level of digital economy of a county, the more rural tourism entrepreneurship it will have in the 12th and 13th Five-Year Plan periods. In particular, an increase of 1 in the Digital Infrastructure Index (DII) increases the ratio of the number of entrepreneurship between 2011 and 2020 to the stock of rural tourism in 2018 (hereinafter referred to as “entrepreneurship ratio”) by 0.433 percentage points. The Rural Economy Digital Index (REDI) increases by 1, and the Entrepreneurship Ratio increases by 0.438 percentage points. An increase of 1 in the Rural Governance Digital Index (RGDI) increases the entrepreneurship ratio by 0.27 percentage points. Among the three independent variables, the rural economy digital index has the largest impact. Thus, the understanding and application of the digital economy and the digital transformation of the industry chain by enterprises have the strongest effect on promoting entrepreneurial activities.

In column B of Table 4, we find that only two digital economy index have a negative and significant relationship with “Exit of entrepreneurship” (EOE): the digital infrastructure index (DII) and the Rural Economy Digitalization Index (REDI). The economic significance is that the higher the level of digital economy in a county, the lower the number of entrepreneurial exits in the 12th and 13th Five-Year Plan periods. In particular, an increase of 1 in the Digital Infrastructure Index decreases the ratio of the number of entrepreneurial exits between 2011 and 2020 to the stock of farmhouses in 2018 (hereinafter referred to as the “exit rate”) by 0.233 percentage points. The rural economy digitalization index increases by 1, and the “exit rate” decreases by 0.324 percentage points. The effect of rural governance digital index (RGDI) on the “exit rate” is negative but statistically insignificant. We can conclude that the understanding and application of the digital economy and the digital transformation of the industry chain can help entrepreneurs to stay in the market for a long time.

In column B of Table 4, the “Rural Economy Digitalization Index” (REDI) is negatively associated with “Exit of entrepreneurship” (EOE) and is statistically significant at the 0.001 level. The economic significance is that an increase of 1 in a county’s “Rural Economy Digitalization Index” (REDI) decreases the number of entrepreneurial exits in this county by 0.324. “Digital Infrastructure Index” (DII) and “Rural Governance Digitalization Index” (RGDI) have no significant effect on entrepreneurial exits, and digital infrastructure index is weakly positively associated with “Exit of entrepreneurship” (EOE).

In column C of Table 4, we use the average life cycle of exited entrepreneurship (ELC) as the dependent variable, and only the effect of the “Rural Economy Digitization Index” (REDI) is significant and positive. The economic significance is that the higher the level of digitalization of the rural economy in a county, the longer the survival time of tourism start-ups in this county in the 12th and 13th Five-Year Plan periods. An increase of 1 in the index increases the survival time of tourism start-ups by 0.211 months.

Through columns B and C, we can conclude that the understanding and application of the digital economy and the digital transformation of the industry chain can help entrepreneurs to stay in the market for a long time.

### Digital economy and product differentiation innovation: Mediation mechanism

Product innovation plays the role of a mediating mechanism and is the most important theoretical finding of this paper. The digital economy stimulates the emergence of innovative behavior and the formation of markets characterized by product differentiation, which in turn stimulates entrepreneurial activity. Because the process of product innovation for entrepreneurship has been verified by the product matching model, we focus on testing the impact of the digital economy on product differentiation innovation. The econometric model continues using equation (6), the difference being that the dependent variable changes and we obtain results using zero-inflated Poisson regression of the counting model Table 5 shows the regression results.

**Table 5:** Baseline regression results for digital economy and innovation.

	D: OPMV			E: CBLV			F: RTKV		
DII	0.005 (0.010)			0.004 (0.01)			0.018 (0.016)		
REDI		0.024^*^ (0.01)			0.01^**^ (0.009)			0.025^**^ (0.01)	
RGDI			0.011 (0.011)			0.002 (0.007)			0.016 (0.012)
Wald Chi^2^(11)	67.33	94.75	77.26	38.74	37.23	38.33	42.46	45.52	41.77
Prob > Chi^2^	0.000	0.000	0.000	0.000	0.000	0.000	0.000	0.000	0.000
Pseudo *R*^2^	0.123	0.134	0.126	0.051	0.053	0.050	0.096	0.101	0.094
*p*-Value	0.634	0.074	0.318	0.713	0.397	0.767	0.274	0.023	0.159
obs	150	150	150	150	150	150	150	150	150

* indicates significant at the 0.05 level.

** indicates significant at the 0.01 level.

All test models include control variables and provincial fixed effects.

In column D of Table 5, the coefficients of all three digital economy indicators are positive, but only the effect of the “Rural Economy Digitalization Index” (REDI) is statistically significant. The economic significance is that an increase of 1 in REDI is associated with an increase of 0.024 in the number of “National One Village, One Product Model Villages (Leisure Tourism)” (OPMV) in that county.

In column E of Table 5, the coefficients of all three digital economy indicators are also positive, but only the effect of the “Rural Economy Digitalization Index” (REDI) is statistically significant. The economic significance is that an increase of 1 in REDI is associated with an increase of 0.01 in the number of “China’s Beautiful Leisure Villages “(CBLV) in that county.

In column F of Table 5, the coefficients of all three digital economy indicators are also positive, but only the effect of the “Rural Economy Digitalization Index” (REDI) is statistically significant. The economic significance is that an increase of 1 in REDI is associated with an increase of 0.025 in the number of “National Key Villages of Rural Tourism “(RTKV) in that county.

We found that among the three digital economy indicators, the Rural Economy Digitalization Index (REDI) has the largest and statistically significant impact on entrepreneurship and innovation. Therefore, we believe that entrepreneurial activity in this region will be boosted if companies better understand and use digital technologies and use data as an important input factor for production and sales, as well as if the industry chain changes business models through digital technologies.

Due to the important role of the “Rural Economy Digitalization Index” (REDI), we use it as the only independent variable in the endogeneity analysis and robustness tests below, and we use “Increased In Entrepreneurship” (IIE) as the only dependent variable.

### Endogenous

The “Rural Economy Digitization Index” may be influenced by the size of the tourism market and entrepreneurial activity that already exists in a county; therefore, the independent variable and dependent variable appears to be mutually causal and lead to biased coefficients of the estimates. We use instrumental variables to address this issue, and we select the “Transportation Distance” between each county government site and the Hangzhou city government site as the instrumental variable. The reasons are as follows: first, Hangzhou is considered the birthplace of the digital economy in the Yangtze River Delta region, and according to the first law of geography, entrepreneurs in counties closer to Hangzhou are more strongly influenced by Hangzhou’s digital economy thinking, such that the “Transportation Distance” is negatively correlated with the dependent variable. Secondly, except for the counties under the jurisdiction of Hangzhou, the markets of other counties are the neighboring cities in the immediate vicinity, and Hangzhou has little influence on their tourism development and naturally little influence on their entrepreneurial activities, so the “Transportation Distance” is not related to the dependent variable. “Transportation Distance” can only affect entrepreneurial activity by influencing the level of digitalization of the rural economy in other counties. In this paper, we use the minimum commuting time by car as the value of the instrumental variable and use two-stage least squares to test for endogeneity. The test results are shown in Table 6.

**Table 6:** Endogeneity test: digital economy and entrepreneurship.

G: Results of the first phase		H: Results of the second phase		I: Exogeneity test		J: Weak instrumental variable	
	**REDI**	**REDI(re)**	**IIE**				
Transportation-distance	−0.065^***^ (0.011)		5.875^***^ (1.734)	Durbin (score) Chi^2^(1):	6.786	Partial-*R*^2^	0.196
				*p*-Value	0.009	*F*(1,140)	34.212
Control	Yes	Control	Yes	Wu–Hausman *F*(1,139)	6.583	Prob > *F*	0.000
*F*(9,140)	14.29	Wald Chi^2^(9)	36.0.30	p-Value	0.011	Minimum eigenvalue statistic	34.212
Prob > *F*	0.000	Prob > Chi^2^	0.000			2SLS size of nominal 5% Wald test	16.38
*R* ^2^	0.4788	*p*-Value	0.001				
*p*-Value	0.000	obs	150				
obs	150						

*** indicates significant at the 0.001 level.

1. The first stage is the regression of the endogenous independent variable on the instrumental variable, and the second stage is the regression of the dependent variable on the independent variable with the residuals removed. 2. The reference value used in the “2SLS Size of nominal 5% Wald test” is the key value at the 10% position.

Column G of Table 6 shows the results of the first stage regression. After controlling for confounding variables in the baseline regression, the instrumental variable has a negative relationship with the “Rural Economy Digitalization Index” (−0.065) and is statistically significant at the 0.001 level. The economic significance is that the further a county is from Hangzhou in terms of commuting distance, the lower its level of rural economic digitalization.

In Column G of Table 6, we remove the residuals of the endogenous explanatory variables using instrumental variables and the new explanatory variable “REDI (re)” has a positive coefficient (5.875) that is statistically significant at the 0.001 level. The economic significance is that the “Rural Economy Digitization Index does” increase the number of entrepreneurship in a county, corresponding to the results of the baseline regression in Table 4.

In column I of Table 6, the Hausman test was adopted for the exogeneity test. The results of the $\chi_{J}^{2}$-test (6.786) and the *F*-test (6.583) are reported in the table, and the *p*-values indicate that the rural economy digitization index is an endogenous variable. Therefore, it is justified to use instrumental variables.

Column J of Table 6 shows the weak instrumental variable tests. We took two approaches, the summary of the first stage results and the Stock/Yogo weak instrumental variable test, respectively. The summary of the first stage regression shows that excluding the effects of other exogenous factors, the instrumental variables still explain 19.6% of the variation in the dependent variable with an F-statistic value greater than 10 and significant. Using simple criteria, we conclude that there are no weak instrumental variables. In the Stock/Yogo weak instrumental variable test, the value of “Minimum eigenvalue statistic” (which is 34.21) is significantly greater than the value of “2SLS Size of nominal 5% Wald test “(which is 16.38). Using the complex criteria, we conclude that there is no weak instrumental variable.

Further, we proceed to test whether endogeneity interferes with the impact of the “Rural Economy Digitization Index” on a county’s tourism product innovation. The test we use remains the same two-stage least squares method. Because of the large number of mediating variables and to save space, we only present the second-stage results and some key statistical test information in Table 7.

**Table 7:** Endogeneity test: Digital economy and product innovation.

	K: OPMV	L: CBLV	M: RTKV
REDI(re)	0.169^**^ (0.053)	0.212^**^ (0.059)	0.183^**^ (0.055)
Durbin (score) Chi^2^(1):	8.954	9.907	9.112
*p*-Value	0.000	0.000	0.000
Minimum eigenvalue statistic	39.99	51.88	43.66
obs	150	150	150

** indicates significant at the 0.01 level.

All test models include control variables and provincial fixed effects.

We removed the residuals of the endogenous explanatory variable (REDI) using instrumental variables and obtained a new explanatory variable [REDI (re)]. Table 7 show that all three regression coefficients are positive and statistically restricted at the level of 0.01, further confirming the fact that the higher the “Rural Economy Digitization Index,” the more counties have “National One Product One Village Characteristic Village”(OPMV), “China Beautiful Leisure Village”(CBLV) and “National Rural Tourism Key Villages”(RTKV), as well as, the higher the innovation of tourism products in this county.

### Testing for spatial effects

We argue that spatial correlation may interfere with the impact of a county’s digital economy on tourism product innovation and entrepreneurship in that county. The reasons are as follows: first, tourism landscapes are the basis of tourism, and some large-scale tourism landscapes (e.g., mountains, forests, and rivers) cover multiple counties, leading to a correlated tourism economy in the immediately neighboring counties. Second, neighboring counties generally share the same culture and frequent social exchanges, leading to similar market behavior characteristics of residents in these areas, as well as their innovative and entrepreneurial behaviors. Finally, visitors to one county often come from another region, so the digital economy of another region may affect tourism in that county. In summary, the data may be spatially dependent and the baseline regression results may be unreliable. Following the approach of Deller et al. (2022), this paper controls for spatial dependence through a spatial weight matrix. Table 8 show the new regression results.

**Table 8:** Control space correlation.

	N: IIE	P: OPMV	Q: CBLV	S: RTKV
REDI	0.371^**^ (0.104)	0.022^*^ (0.010)	0.008^*^ (0.004)	0.019 (0.009)
*p*-Value > Chi^2^(9)	0.006	0.011	0.111	0.052
Rho	0.016	0.103	1.107	0.433
Sigma	11.08	13.59	17.21	14.88
(Buse 1973) *R*^2^	0.139	0.196	0.265	0.203

* indicates significant at the 0.05 level.

** indicates significant at the 0.01 level.

Column N of Table 8 can be compared with column A of Table 4, and columns P, Q, and S can be compared with Table 5. We find that when controlling for spatial correlation, the coefficient of the Rural Economy Digitalization Index (REDI) becomes smaller but still positive and statistically significant at the 0.05 level. We can still get the conclusion that the digitalization index of rural economy promotes tourism product innovation and tourism entrepreneurship.

### Sensitivity analysis (omitted variable analysis)

**Figure 2:**
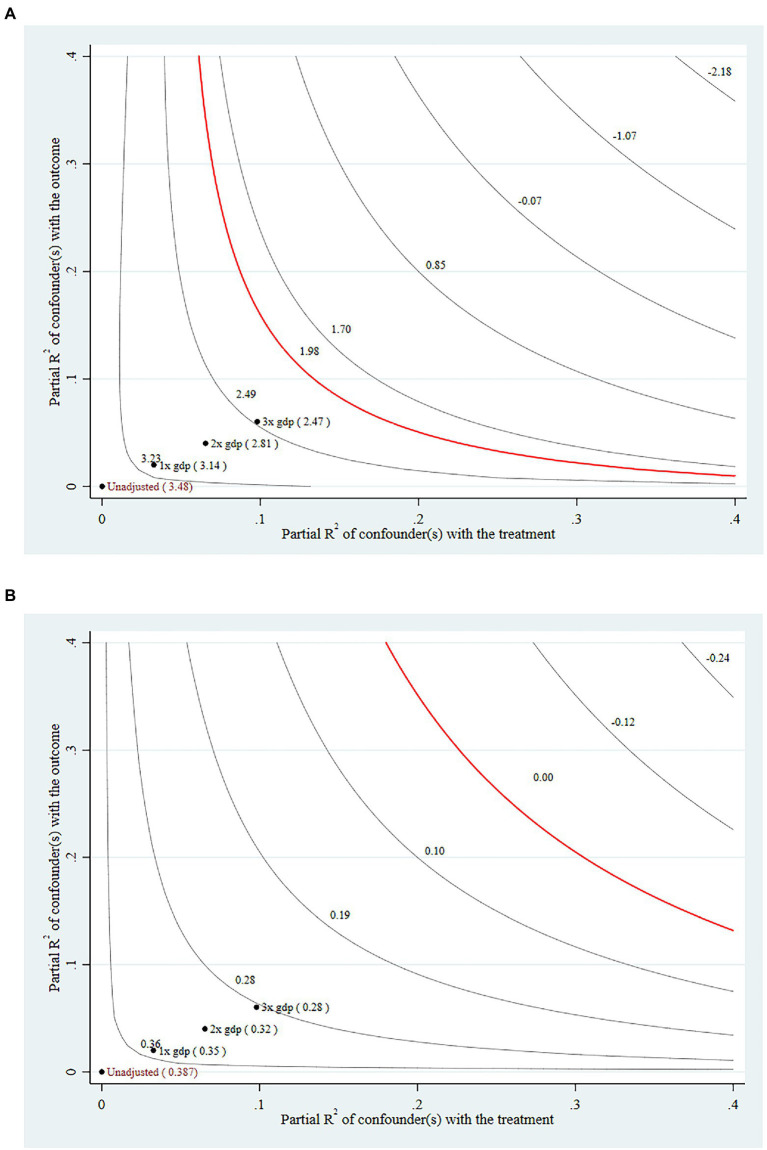
**(A)** The red line indicates the threshold at which the significance of the coefficients can be overridden, and when we successively add omitted variables with explanatory power, the threshold is not breached. The fact that the coefficients are significant in the baseline model is robust. **(B)** The red line indicates the threshold at which a positive coefficient can be overturned, and when we successively add omitted variables with explanatory power, the threshold is not breached. The fact that the coefficients are positive in the baseline model is robust.

A problem that tends to arise in cross-sectional analysis is the bias of coefficients due to omitted variables. We finally use sensitivity analysis to test the possibility of this problem. The test is if there are omitted variables, how strong of an explanatory power does the omitted variable need to have to overturn the results of the baseline regression? Or how robust are the baseline regression results in the worst case scenario (where the omitted variable contains all the remaining variance of the explained variable)? To accomplish this task, we set a dummy omitted variable and assume that this omitted variable has three times the effect on the dependent variable than GDP (we use “GDP” for comparison because GDP is the most basic measure of the level of economic activity in a county). To save space, our focus remains on the relationship between the “Rural Economy Digitalization Index” and the “Increase In Entrepreneurship,” the causal relationship of most interest in this paper. The results of the analysis are shown in Figure 2A,B.

In Figure 2A, the statistical significance of the effect of the “Rural Economy Digitalization Index” on the “Increase In Entrepreneurship” does not change in any way when we include the potential omitted variable of three times the explanatory power of GDP. In Figure 2B, the direction of the effect of the “Rural Economy Digitalization Index” on the “Increase In Entrepreneurship” does not change in any way when we include a potential omitted variable with an explanatory power 3 times that of GDP. Therefore, we consider the results obtained from the benchmark regression to be robust.

## Conclusions and recommendations

This paper examines the impact of the Digital Economy on rural entrepreneurial activity and extends the perspective to a specific rural industry—tourism. We obtain the following important conclusions: (1) The Digital Economy promotes tourism entrepreneurial activity in rural areas, which is consistent with most of the literature on the digital economy (or digital technology). (2) A new mechanism for the Digital Economy to act on entrepreneurial activities was discovered—product differentiation innovation. The digital economy directly reduces the cost of innovation and thus stimulates innovative behavior, and a market characterized by product innovation will spontaneously stimulate more entrepreneurial activities. (3) The Digital Economy includes multiple dimensions, and it is the “Rural Economy Digitalization Index” that can work through intermediary mechanisms, other dimensions of the digital economy do not promote product innovation.

Based on the findings in (2) and (3) in the previous paragraph, we propose two policy recommendations: First, in the future, government investment could be directed away from infrastructure and toward the innovative capabilities of entrepreneurs. For example, improving entrepreneurs’ understanding and application of digital technologies, promoting the digital transformation of business models in the tourism chain, and increasing the input of data elements in the production process. Second, existing policies to support funding for entrepreneurial activity may lead to market failures, i.e., short business life cycle or (and) high business exit. We believe that the government should focus in the future on how to provide a good market environment for operators’ innovative behavior, thus addressing the current widespread lack of innovation and leaving how to increase entrepreneurship to market mechanisms.

The limitations and shortcomings of this paper are reflected in the fact that we do not consider whether the personal traits of entrepreneurs influence the mechanisms of action of the digital economy. This new study requires more micro-level data, and in the future we will try to use innovation survey data to study the innovation choices and entrepreneurial quality of existing entrepreneurs.

## Data availability statement

The original contributions presented in the study are included in the article/supplementary material, further inquiries can be directed to the corresponding author.

## Author contributions

GT is responsible for the topic of the manuscript and the structure of the manuscript. FR is responsible for writing the manuscript and data analysis. JZ is responsible for finding the data. All authors contributed to the article and approved the submitted version.

## Funding

This project was supported by the Science and Technology plan projects of Zhejiang Province (2021C35096), Annual Project of Zhejiang Provincial Philosophy and Social Science Planning (22NDJC056YB), and Philosophy and Social Science Foundation of Hangzhou (M22JC089).

## Conflict of interest

The authors declare that the research was conducted in the absence of any commercial or financial relationships that could be construed as a potential conflict of interest.

## Publisher’s note

All claims expressed in this article are solely those of the authors and do not necessarily represent those of their affiliated organizations, or those of the publisher, the editors and the reviewers. Any product that may be evaluated in this article, or claim that may be made by its manufacturer, is not guaranteed or endorsed by the publisher.
